# Metagenomic Detection of Viral Pathogens in Spanish Honeybees: Co-Infection by Aphid Lethal Paralysis, Israel Acute Paralysis and Lake Sinai Viruses

**DOI:** 10.1371/journal.pone.0057459

**Published:** 2013-02-27

**Authors:** Fredrik Granberg, Marina Vicente-Rubiano, Consuelo Rubio-Guerri, Oskar E. Karlsson, Deborah Kukielka, Sándor Belák, José Manuel Sánchez-Vizcaíno

**Affiliations:** 1 Department of Biomedical Sciences and Veterinary Public Health (BVF), Swedish University of Agricultural Sciences (SLU), Uppsala, Sweden; 2 The OIE Collaborating Centre for the Biotechnology-based Diagnosis of Infectious Diseases in Veterinary Medicine, Uppsala, Sweden; 3 Animal Health Department, Faculty of Veterinary, Complutense University of Madrid, Madrid, Spain; 4 SLU Global Bioinformatics Center, Department of Animal Breeding and Genetics (HGEN), SLU, Uppsala, Sweden; 5 Department of Virology, Immunobiology and Parasitology, VIP, National Veterinary Institute (SVA), Uppsala, Sweden; Columbia University, United States of America

## Abstract

The situation in Europe concerning honeybees has in recent years become increasingly aggravated with steady decline in populations and/or catastrophic winter losses. This has largely been attributed to the occurrence of a variety of known and “unknown”, emerging novel diseases. Previous studies have demonstrated that colonies often can harbour more than one pathogen, making identification of etiological agents with classical methods difficult. By employing an unbiased metagenomic approach, which allows the detection of both unexpected and previously unknown infectious agents, the detection of three viruses, Aphid Lethal Paralysis Virus (ALPV), Israel Acute Paralysis Virus (IAPV), and Lake Sinai Virus (LSV), in honeybees from Spain is reported in this article. The existence of a subgroup of ALPV with the ability to infect bees was only recently reported and this is the first identification of such a strain in Europe. Similarly, LSV appear to be a still unclassified group of viruses with unclear impact on colony health and these viruses have not previously been identified outside of the United States. Furthermore, our study also reveals that these bees carried a plant virus, Turnip Ringspot Virus (TuRSV), potentially serving as important vector organisms. Taken together, these results demonstrate the new possibilities opened up by high-throughput sequencing and metagenomic analysis to study emerging new diseases in domestic and wild animal populations, including honeybees.

## Introduction

In recent years, the world’s population of honeybee (*Apis mellifera*), the main insect pollinator in the USA and Europe, has been decreasing. This loss of bee population has been called Colony Collapse Disorder (CCD), defined as the disappearance of bees from the beehives without any dead bees around the affected hives, presence of abundant breeding cells, pollen, and honey despite a small population of adult bees and without any characteristic symptom of disease [1]. The importance of this phenomenon stems not only from the large direct losses of honey-producing countries, of which Spain has the largest honey production in the European Union (EU) and EU ranks third in the world honey production CCD may also cause indirect losses for a lack of crop pollination, some of them very important in Spain such as citrus crop. To illustrate the serious effects of honeybee decline, which is the most efficient pollinator of most crops [2], the global value of insect pollination has been estimated at US$ 212 billion (€153 billion), which represents about 9.5% of the total value of agricultural production. Specifically, the value of insect pollination to agriculture for EU25 is US$ 19.8 billon (€14.2 billion) [3]. As a result, the CCD phenomenon has become a growing concern for governments and international organizations, which have led to increased investments in terms of research on its origin.

The manifestations of increased honeybee mortality and decline of managed hives due to unclear diseases, such as CCD, have to a great extent been associated with viral infections [4], [5]. At least 18 different viruses with the ability to infect honeybees have so far been identified [6], [7], and an additional four were recently suggested [8]. Several of them have been demonstrated to have a global spread [9], [10] and colonies frequently suffer from multiple viral co-infections [11]–[14]. In addition, since most of the honeybee viruses tend to persist as covert infections with no obvious symptoms, requiring stress to be activated [15] even seemingly healthy colonies can harbour a variety of potentially harmful viral infections [16], [17]. Among the natural activation factors, especially mite infestations have been correlated with outbreak of disease. The parasitism is believed to induce virus proliferation by causing a general decline in immune capacity of the hosts and mites have also been shown to act as vectors of honeybee viruses [18]–[20].

Regarding the above listed serious problems and losses, there is a high need to investigate the occurrence, emergence and effects of various pathogens in the European honeybee populations, with special regard to known and “unknown”, emerging new viruses and to the combination of various agents in complex infections. For the time being, the direct diagnosis of the various disease forms is based on two main approaches: a) traditional diagnostic methods, such as virus isolation and electron microscopy; b) molecular diagnostic methods, such as PCR and microarrays, among others.

The traditional paradigm of detecting and identifying pathogens relies upon diagnostic tests available for the detection of known agents. This makes it difficult, or impossible, to identify unexpected or novel pathogens by using conventional methods.

Virus isolation has a very powerful diagnostic capacity, considering that this method is able to detect a very wide range of viruses directly, in a single system. However, even this excellent method has many weaker sides, e.g., the inability of many viruses, including honeybee viruses, to replicate in the used cell cultures and cause visible signs of virus replication, such as cytopathic effects.

Molecular diagnostic techniques, such as PCR, isothermal amplification, and microarrays have rapidly been replacing the traditional diagnostic approaches and have opened new alternatives for virus detection and identification. However, even these methods have important weak sides, such as the restricted detection range. The detection range can be improved by the application of wide range microarrays, DNA-chips, however, even these system may fail to detect a wide range of pathogens, for example “unknown”, emerging new viruses. Considering that many of these viruses may cause severe diseases, malfunctions, synergetic effects with other pathogens, may influence the immune system, and many other effects, it is crucial to improve our diagnostic capacities and to extend our detection capacities.

The introduction of viral metagenomics has opened up a new range of possibilities for the improved detection of both known and unknown viruses. These cell culture and nucleotide sequence independent approaches allow the detection of a very wide range of viruses and other pathogens and they have the capacity to determine the entire infectious flora in different host species. Furthermore, viral metagenomics is able to shed light not only of the presence of various infectious agents, but also on the biodiversity of the detected viruses, bacteria and other infectious agents. This enables us to achieve a better understanding of emerging novel diseases and the complex infection biology of various disease complexes. The comprehensive metagenomic techniques, such as high-throughput nucleotide sequencing, have the potential to detect the full spectrum of emerging new pathogens, including novel viruses and fastidious bacteria, as demonstrated and reviewed [21]–24. The *OIE Collaborating Centre for the Biotechnology-based Diagnosis of Infectious Diseases in Veterinary Medicine* in Uppsala, Sweden has established skills and state-of-the-art facilities for the metagenomic detection of various known and unknown viruses, such as novel bocaviruses, Torque Teno viruses, astroviruses and other infectious agents [25]–[27].

Given the unclear diagnosis of many honeybee viral diseases, the frequent covert infection of these viruses and the high prevalence of multiple viral co-infections, we hypothesize that a metagenomic approach should be particularly useful to find various potentially causative agents in the bee colonies. This has also been demonstrated by previous studies aimed at characterizing the microflora, or microbiome, of the honeybee in search of microbial agents involved in CCD [4], [8]. In this study, honeybees from Spain were investigated using a high-throughput sequencing approach to identify all potential etiological agents.

## Materials and Methods

### Specimens

The sample of honeybees (*Apis mellifera*) was collected with the owners’ permission from one colony belonging to one apiary of 25 commercial hives located in Los Arcos, Navarre, North of Spain. The colony was sampled by the veterinary services due to lack of vitality of adult worker honeybees and unusual depopulation, especially in the brood frames. Furthermore, symptoms compatible with CCD such as drastically reduced adult population in presence of abundant food and breeding were observed. There were no specific symptoms compatible with viral diseases. The sample consisted of approximately 50 adult worker bees from inside and outside the hives to ensure the presence of young and adult bees. The bees were collected in sterile containers and frozen until delivered to the Department of Animal Health at the Complutense University of Madrid for routine testing with standard RT-PCR assays for identification of common bee viruses. Homogenates were manually prepared from 20 whole bees, in a 30 ml Wheaton glass homogenizer containing 6 ml of sterile phosphate-buffered saline (1×PBS).

The sample was analyzed by amplification of virus-specific nucleic acid for the presence of seven honeybee viruses: Deformed Wing Virus (DWV), Black Queen Cell Virus (BQCV), Sacbrood Bee Virus (SBV), Acute Bee Paralysis Virus (ABPV), Chronic Bee Paralysis Virus (CBPV), Kashmir Bee Virus (KBV) and Israeli Acute Paralysis Virus (IAPV). One step real time RT-PCRs based on SYBR-Green dye were carried out, following previously described protocols [13], . The sample tested positive for IAPV and the viral load was estimated to be 7.5×10^4^ genome equivalent copies (GEC) per bee.

### Sample Preparation and Nucleic Acid Isolation

The homogenates were centrifuged at 4.000 rpm for 10 min and the collected supernatants were syringe-filtered through disposable 0.45 µm PVDF filters (Millipore). Aliquots of 200 µl supernatant in a final concentration of 1xDNase buffer were nuclease treated with 400 U/ml DNase I (Roche Applied Science) and 8 µg/ml RNase A (Invitrogen) at 37°C for 2 h. DNA was extracted using the QIAamp DNA Mini Kit (Qiagen) according to the manufacturer’s spin protocol for blood and body fluid. RNA was isolated using TRIzol LS Reagent (Invitrogen) and further purified using the RNeasy mini kit (Qiagen) according to the manufacturer’s instructions.

### Tag Labeling and Random Amplification

Extracted DNA and RNA were separately labeled with an identical sequence tag contained in the primer FR26RV-N (GCCGGAGCTCTGCAGATATCNNNNNN) [32]. For the labeling of DNA, 10 µl of template was mixed with 1.5 µl 10× NEBuffer 2, 1.5 µl dNTPs (10 mM of each), and 2 µl FR26RV-N (10 mM). The mixture was denatured at 94°C for 2 min and chilled on ice before the addition of 0.5 µl (2.5 U) 3′–5′ exo- Klenow DNA polymerase (New England Biolabs). The initial extension at 37°C for 1 h was followed by an identical second cycle, starting with denaturation as above, after which the enzyme was inactivated by heating at 75°C for 10 min. The synthesis of the sequence tagged cDNA was prepared by adding 1.5 µl dNTP (10 mM of each) and 2 µl FR26RV-N (10 mM) to 10 µl of RNA template. The mixture was incubated at 65°C for 5 min and chilled on ice before the addition of 4 µl 5× First-Strand buffer (Invitrogen), 1 µl DTT (100 mM), 1 µl (40 U) RNaseOUT (Invitrogen) and 1 µl (200 U) Superscript III reverse transcriptase (Invitrogen). The RT reaction was incubated at 25°C for 5 min, 50°C for 1 h and 70°C for 15 min, after which it was chilled on ice. Second-strand synthesis was performed by adding 0.5 µl (2.5 U) 3′–5′ exo- Klenow DNA polymerase (New England Biolabs) and incubate at 37°C for 1 h. A final incubation at 75°C for 10 min inactivated the enzyme.

Amplification was performed by PCR using the complementary primer FR20RV (GCCGGAGCTCTGCAGATATC) [32]. Each 50 µl reaction was carried out with 2.5 µl of labeled template and 0.5 µl (2.5 U) Ampli-Taq Gold DNA polymerase (Applied Biosystems) in a final concentration of 1x GeneAmp PCR buffer 2 (Applied Biosystems), 0.2 mM dNTPs, 2.5 mM MgCl_2_, and 0.8 µM FR20RV. The thermal cycling was initiated with a denaturation step at 95°C for 10 min, followed by 40 cycles of 95°C for 1 min, 58°C for 1 min, 72°C for 1 min, and a final extension at 72°C for 10 min. PCR products were purified with the QIAquick PCR purification kit (Qiagen), both before and after the tag sequence was removed with EcoRV (New England Biolabs), according to the manufacturers’ instructions. The final products were checked on agarose gel and quantified using a NanoDrop ND-100 spectrophotometer (NanoDrop Technologies).

### Library Preparation and Sequencing

The library preparation and sequencing were performed at the SNP&SEQ Technology Platform in Uppsala. Briefly, the amplification products were pooled and separated into two size fractions of approximately 250–400 bp and 400–550 bp, respectively. Each fraction was labeled, without any further fragmentation, with an indexing sequence using GS FLX Titanium Rapid Library MID Adaptors (Roche) and sequenced on 1/16 of a GS FLX Titanium PicoTiterPlate (Roche/454 Life Sciences) according to the manufacturer’s protocol.

### Data Handling and Bioinformatics

The sequence data, trimmed of adaptor regions and in Standard Flowgram Format (SFF), were combined into a single FASTA and quality file using the sff_extract python application distributed with the MIRA software package (http://sourceforge.net/projects/mira-assembler). All sequence reads were then assembled using MIRA [33] with the standard settings for *de novo* assembly of 454 data.

Taxonomic classification of unassembled sequence reads was enabled by BLASTN and BLASTX searches against local copies of NCBI’s nucleotide and protein databases using NCBI’s blastall program [34] with default settings. The resulting outputs were committed into MEGAN 4 [35] with the NCBI taxonomy data for assigning taxa. Each sequence read was placed on a node in the NCBI taxonomy according to the lowest common ancestor (LCA) based on a subset of the best scoring BLAST matches. The parameters for the LCA algorithm were: Min support 5, Min Score 65, Top Percent 10, and Min complexity 0.3. Resulting trees were explored for host genome, bacterial content and viral community.

Evaluating the taxonomic data for potential pathogens, candidate reference genomes were identified and retrieved from GenBank in FASTA format. Alignment of matching contigs from the whole assembled dataset against the nucleotide sequences of the reference genomes were performed using the CodonCode Aligner software (CodonCode Corporation). This allowed analysis of similarities and visualisation of gaps.

### Confirmation and Retrieval of Near Full Genome Sequences

Based on the results from the alignments, PCR primers were designed to confirm the presence of viruses in the original material and to close gaps using the Primer3 program [36]. Total RNA was extracted from filtered homogenate as above, but without nuclease treatment, and cDNA was generated using the Superscript III first-strand synthesis system (Invitrogen) with random hexamers according to the manufacturer’s instructions. Products with an expected length shorter than 1.500 bp were amplified with an AmpliTaq Gold-based PCR protocol (Applied Biosystems), while the Phusion Hot Start II High Fidelity DNA Polymerase system (Thermo Scientific) was used for longer fragments. The amplified products were size-separated on an agarose gel, purified with the QIAquick Gel Extraction Kit (Qiagen), and sent for sequencing (Macrogen Europe). The obtained sequences were incorporated into the alignments against the reference genomes using the CodonCode Aligner software (CodonCode Corporation). Longer distances were covered by iterative primer walking. The resulting viral sequences reported in this paper have been deposited in GenBank.

### Comparison between Sequences and Phylogenetic Analysis

Direct comparisons between two sequences on a nucleotide and amino acid level were performed by using the NCBI’s BLAST 2 sequences tool [37]. To enable multiple species and strain comparisons, annotated sequences were retrieved from GenBank in FASTA format. While the complete sequences were directly used for phylogenetic analysis, the partial were aligned against a reference genome using the CodonCode Aligner software (CodonCode Corporation). By using the genomic region with the highest number of overlaps, a maximum number of strains were allowed to be compared. The analysis of the phylogenetic relationships were conducted in MEGA 5 [38] using Clustal W [39] to align the sequences and the Maximum Likelihood method with 1.000 bootstrap replicates to generate the trees [40]. The Kashmir Bee Virus (KBV), which is a *Dicistroviridae* member genetically closed to IAPV [41], was used as outgroup.

## Results

### Sequence Data and Assembly

GS FLX Titanium sequencing (Roche/454 Life Sciences) of the nuclease treated and amplified sample returned 161.170 reads for the short fraction (250–400 bp) and 80.790 for the long (400–550 bp). For de novo assembly using MIRA, both fractions were combined resulting in a total of 241.960 reads and 54,98 Mbp of sequence data. The assembly generated 6.350 contigs, ranging in length from 40 to 2.945 bp with mean length 393,3 bp and an average GC content of 51,1%.

### Taxonomic Classification

**Figure 1:**
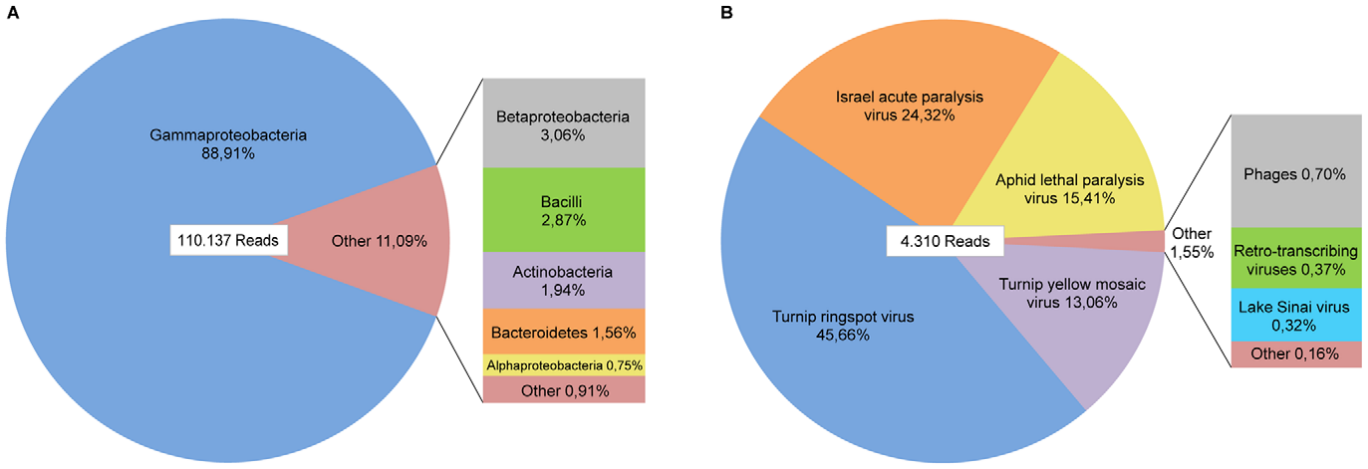
Taxonomic distributions. The distribution of sequence reads within the taxonomic groups of (a) bacteria, and (b) viruses. The taxonomic assignment was performed based on the BLASTN search results using the MEGAN 4 software with the following LCA settings: Min support 5, Min Score 65, Top Percent 10, and Min complexity 0.3.

The assignment of unassembled sequence reads to taxa based on BLASTN results revealed bacteria to be the largest taxonomic group. Most of the 110.137 reads in this group mapped to Gammaproteobacteria, but the presence of Alpha- and Betaproteobacteria, Bacilli (Firmicutes), Actinobacteria, and Bacteroidetes were also indicated, see Figure 1a. No bacterial pathogens, such as *Paenibacillus larvae*, *Melissococcus plutonius* or *Spiroplasma* were detected. Thus, the observed bacterial diversity is similar and in agreement with results from metagenomic studies aimed at characterizing the normal gut flora of honey bees [42], [43]. Among the reads in Eukaryota, 918 corresponded to the host organism *Apis mellifera* and related species, which constitute only 0,38% of the total amount of reads. This indicates that most of the host nucleic acid was removed during the sample preparation step. Furthermore, no matches against mites, such as *Varroa destructor*, or pathogenic fungi, such as certain members of *Nosema*, were detected in the eukaryotic group. A total of 4.310 reads were taxonomically assigned to viruses, the majority belonging to ssRNA viruses. Even though we used a relatively low threshold for allowing assignment, a large number of reads still displayed too short homologies to be uniquely defined and were classified as “Not assigned”. In addition, for 2.194 reads, no hits were found in the NCBI database, and 21.961 were disregarded due to low complexity of the reads. Remaking the taxonomic assignment based on BLASTX searches resulted in a similar distribution as described, the main difference being that approximately 10% of the reads were moved from the not assigned group into bacteria. Even so, no bacterial pathogens were identified.

### Detected Viral Genomic Sequences

As illustrated in Figure 1b, most of the sequence reads in the virus group were divided between four viruses. While both ALPV and IAPV are members of the *Dicistroviridae* family, Turnip Ringspot Virus (TuRSV) and Turnip Yellow Mosaic Virus (TYMV) are plant-infecting viruses belonging to the families *Secoviridae* and *Tymoviridae*, respectively. Low numbers of reads with similarities to Lake Sinai Virus (LSV), retro-transcribing viruses and bacteriophages were also identified.

For each of the specific viruses, a best matching candidate reference genome was retrieved to enable direct comparisons against all assembled contigs. The resulting alignments revealed regions of high sequence coverage and the total numbers of contigs and reads for each virus are summarized in Table 1. In general, the number of sequence reads for a particular virus is proportional to its abundance in the investigated sample. However, the size of the genome and similarity with existing reference genomes can be confounding factors. This makes it hard to judge the lower amount of reads for LSV.

**Table 1:** Identified ssRNA viruses with family classifications and numbers of aligned sequences.

Virus	VirusFamily/Taxa	Reads	Contigs
Aphid lethal paralysis virus(ALPV)	*Dicistroviridae*	664	16
Israel acute paralysis virus(IAPV)	*Dicistroviridae*	1.048	7
Lake Sinai virus (LSV)	Unclassified	14	1
Turnip ringspot virus (TuRSV)	*Secoviridae*	1.968	14
Turnip yellow mosaic virus(TYMV)	*Tymoviridae*	563	1

Regarding the indicated presence of retro-transcribing viruses, two contigs were generated and both displayed a high degree of similarity with Moloney murine leukemia virus and Xenotropic Murine Leukemia Virus (XMLV). However, attempts to amplify a larger fragment spanning the gap between the contigs by PCR failed to generate a product (data not shown). Given the dubious findings of XMLV in many different biological samples [44], it is likely that the findings indicate the presence of contaminants [45] rather than a gammaretrovirus.

### A Virus Similar to Aphid Lethal Paralysis Virus (ALPV)

The 16 contigs displaying similarity with ALPV were distributed in four non-overlapping clusters, spanning a total of 1.469 nt, as demonstrated by alignment against the first complete ALPV genome that was published (GenBank AF536531). Using the consensus sequences of the clusters as starting points, PCR primers were designed in order to fill the gaps by Sanger sequencing and longer distances were bridged by primer walking. This resulted in a single 9.327 nt sequence (GenBank JX045858) covering approximately 95% of the ALPV genome. While the nucleotide homology with the reference genome, as shown by BLAST, was 82%, the recovered sequence displayed an even closer resemblance, 96%, to the recently published ALPV strain Brookings (GenBank HQ871932). Unlike the classical ALPV, which has not yet been associated with infections of honeybees, the Brookings strain was first found in diseased honeybees using a next generation sequencing approach [8]. However, due to the relatively high resemblance with the reference sequence, the question whether or not the new strain constitutes a novel species was left unanswered. A comparison at the protein level, translated DNA against protein sequences, demonstrated that the non-structural polyprotein sequence recovered in this study aligned with 91% amino acid identity to the reference genome and 99% to the Brookings strain. It thus appears likely that we have identified the first European member of a subgroup of ALPV being able to infect honeybees and putatively causing disease.

### A Novel Variant of Israel Acute Paralysis Virus (IAPV)

**Figure 2:**
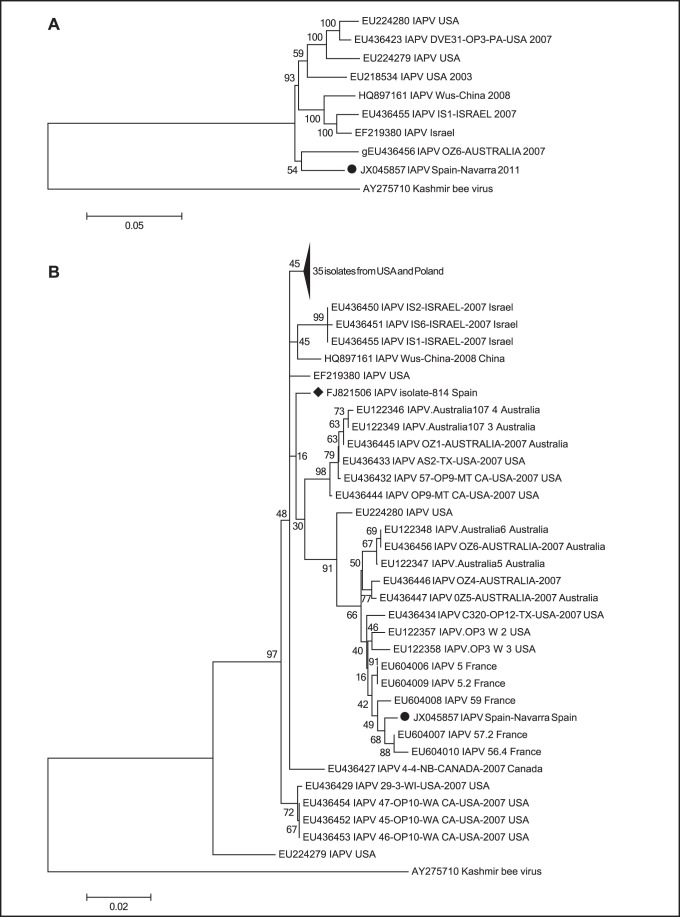
Phylogenetic relationships between strains of IAPV. The trees were based on (a) near full-length genomes, and (b) a 700 nt region (the upper part, containing genetically similar strains from USA and Poland, has been collapsed for clarity). The analyses were conducted in MEGA 5 using Clustal W alignment and the Maximum Likelihood method (bootstrap resampling 1.000 times). The Kashmir bee virus was used as outgroup. The IAPV identified in this study is denoted by (•) and the strain previously identified in Spain with (⧫).

Using the closest blast hit as reference sequence (GenBank EU224279), the seven contigs corresponding to IAPV were aligned into three non-overlapping clusters, covering 816 nt in total. A longer continuous sequence was obtained in a similar manner as above, resulting in a stretch of 8.882 nt (GenBank JX045857) representing approximately 93% of the IAPV genome. The sequence was compared against all publicly available complete IAPV genomes deposit in GenBank. As shown in Figure 2a, the IAPV identified in this study had the highest resemblance to the Australia strain and was more similar to the strains from United States than the ones from Israel and China. In an extended phylogenetic analysis, a stretch of approximately 700 nt from the IGR, containing RNA polymerase and structural polyprotein genes, was chosen to compare the obtained sequence to 67 IAPV strains with publicly available nucleotide sequences. The IGR region of the IAPV genome also contains an independent internal ribosome entry site (IRES) and has proven suitable for the inference of phylogenetic relationships in previous studies [4], [30]. Alignment and clustering indicated that the IAPV identified in this study shared more similarity with strains from France [46], as illustrated in Figure 2b, than the only strain previously identified in Spain (GenBank FJ821506) [47]. At a nucleotide level, the 700 nt of the obtained sequenced displayed 96% similarity to the Spanish strain and 99% to the French. This indicates that the virus is a variant of IAPV with close resemblance to already sequenced strains. Moreover, the high degree of similarity to the strain previously identified in France is correlating well with the geographic proximity.

### A Virus Similar to Lake Sinai Virus (LSV)

A single contig of 631 nt (GenBank JX045859) indicated the presence of a virus similar to LSV, which was only recently discovered [8]. The gene organization of LSV has been described to resemble the genome of Chronic Bee Paralysis Virus (CBPV). However, only a low degree of similarity is retained between LSV and CBPV at the gene level, e.g. the Orf1 genes only display 18% amino acid identity. As demonstrated by blast analysis, the best match for the recovered contig is the Orf1 region of LSV1 (GenBank HQ871931) and LSV2 (GenBank HQ888865), and the nucleotide similarity is 78 and 77%, respectively. Since only one contig was obtained, iterative primer walking could not be applied to obtain a longer sequence. Nevertheless, the presence of the contig sequence in the original sample was verified by PCR and Sanger sequencing. On an amino acid level, a similar comparison revealed that the contig shared 87 and 80% max identity with LSV1 and LSV2, respectively. This indicates that we have made the first observation of a virus within the same group as LSV outside of the United States.

### Turnip Ringspot Virus (TuRSV) & Turnip Yellow Mosaic Virus (TYMV)

Aligning the contigs corresponding to TuRSV against the closest BLASTN match, isolate Toledo segment RNA 1 (GenBank FJ712026), resulted in three non-overlapping clusters with a combined length of 1.169 nt (GenBank JX045854-6). A direct comparison of these clusters with the reference genome revealed that the nucleotide sequence similarity was approximately 90 to 94%. The presence of TuRSV in Spain has previously been established [48], but the published sequence do not overlap with the contigs making a direct comparison impossible (GenBank AJ489259). For TYMV, one contig of 225 bp was generated and it shared 91% nucleotide sequence similarity with its most similar reference genome (GenBank X07441), but only over a stretch of 56 bp in the middle. Since the ends did not show any resemblance with the reference, this could either indicate a new type of TYMV-like virus or an incorrectly assembled contig. As only one short contig of uncertain nature was obtained, we did not proceed to verify this finding. Although likely to be associated with pollen and nectar, honeybees have not explicitly been shown to be a vector for the spread of TuRSV or TYMV.

## Discussion

The high occurrence of co-infections in colonies and honeybees has made it desirable to investigate multiple pathogens when attempting to identify the causative etiological agents in the known and in the recently emerging, “unknown” infectious diseases of the various populations. The detection of known and “unknown” infectious agents has recently been greatly facilitated by the application of metagenomic approaches, exploiting the emergence of high-throughput sequencing techniques, which allows the simultaneous detection and characterization of various microorganisms, including bacteria, viruses, fungi and parasites. Using this type of unbiased metagenomic approach, we here identified and confirmed the presence of three viruses, ALPV, IAPV and LSV, in honeybees from Spain. The existence of a subgroup of ALPV with the ability to infect bees was, together with LSV, only recently reported [8]. Thus, according to our knowledge, this is the first identification of these strains in Europe. Interestingly, this study revealed not only bee viruses in the examined honeybee sample, but also a plant pathogen, TuRSV.

Viruses affecting honeybees have been demonstrated to have a wide spread within the pollinator community. For instance, ABPV has been described to cause covert infections in bumble bee species and KBV has been detected in both bumble bees and wasps [49], [50]. In addition, a more in-depth study to investigate the host range and transmission of common honeybee viruses, such as IAPV and BQCV, found that these viruses are disseminating freely among the pollinators via the flower pollen itself [51]. The same study also revealed that non-*Apis* hymenopteran pollinators near honeybee apiaries affected by IAPV were more likely to carry the virus themselves. There are thus several potential reservoirs for these viruses in nature, which motivates the inclusion of other pollinators when conducting prevalence studies of honeybee viruses.

Until recently, no virus similar to ALPV on the sequence level had been reported in association with honeybees. This was changed, soon after this study began, with the finding of the Brookings strain in the United States, where the initial discovery also enabled the virus to be retroactively detected in honeybee samples from multiple geographic locations collected during the course of several months [8]. The frequent detection made the authors conclude that the virus is not just passively being transported together with forage (nectar and pollen) from flowers shared with other insects. However, they could not associate infection by the Brookings strain to any specific symptoms and called for further studies to determine whether the virus is commensal, using honeybees as vector, or a pathogen.

We here report the first finding and description of an ALPV-like virus in honeybees from Europe. Even though the pathogenicity of ALPV in honeybees thus remain unknown [52], the high sequence similarity to the Brookings strain makes it reasonable to assume that the viruses affect their hosts in a similar manner. Given the frequent occurrence of the Brookings strain in the United States and the discovery of an almost identical virus in Europe, further studies of these ALPV-like viruses are highly motivated. A first step would be to conduct further prevalence studies, both in honeybees as well as related pollinators, to determine distribution and host range, as well as assessing the transmissibility and pathogenicity.

In agreement with the initial correlation of IAPV with CCD [4], it has been demonstrated that specific treatment against IAPV can improve the health of affected colonies [53]. However, causative relationship between IAPV presence and CCD has not been established yet and IAPV has also been reported to exist in many hives with no symptoms of CCD around the world [46], [54], including Spain [55]. The variant of the virus detected in this study shared most similarity with a strain identified in France during an attempt to correlate IAPV with increased colony mortality [46]. Furthermore, the French strain was phylogenetically demonstrated to belong to a sub-lineage comprised of IAPV isolates from apparently healthy bees. In our extended analysis, a similar division was observed, indicating that this IAPV variant might belong to a group of viruses unable to cause overt infections in affected bees and hence, unable to cause CCD alone.

For its part, Lake Sinai virus has only been described in the USA, and this is the first time that it is described in honeybees from Europe. Related to CBPV and the *Nodaviridae* family, the pathogenic implication and the epidemiological relevance remains unknown, despite the fact that LSV could be one of the honeybee viruses previously described by serology or electron microscopy for which no molecular information is available (Bee virus X and Y, Arkansas bee virus and Berkeley bee virus) [8]. More studies should deepen the knowledge and understanding of this honeybee virus.

The finding of TuRSV through the metagenomic approach indicates the presence of this plant virus in the bee samples, but does not provide sufficient information yet on the reason why this plant virus is present in the homogenised bee organs. It is likely that the bees are passively carrying the plant viruses, e.g., in infected pollen particles attached to their body, and it does not seem to be likely that active plant virus replication would occur in the bodies of the bees. However, further investigations are surely required to clarify exactly the infection biology of this scenario. Even these initial results clearly indicate that the honeybees are potentially serving as important vector organisms for transmitting infections from plant to plant.

Given the results, it would have been of interest to perform a follow-up of the sanitary status of the studied colony. Unfortunately, this could not be specifically performed as it was a commercial hive and beekeepers often use new brood frames from healthy colonies to avoid the death of the weak colony. However, the apiary did not present any other problem of depopulation and weakening of colonies in the following years, indicating an improvement of the sanitary status of the colonies.

Taken together, these results serve to illustrate the new possibilities offered by metagenomic analysis, including general amplification, high-throughput sequencing and bioinformatic analysis, to allow investigation of whole virome or microbiome in search of unexpected and previously unknown etiological agents. Our investigation of a colony with CCD-like symptoms revealed that it was not only co-infected by two dicistroviruses and by one unclassified virus, but it also harboured a plant virus. This finding can be compared with standard diagnostic methods, i.e. RT-PCR assays. However, due to their principles and limitations, the PCR methods were able to ascertain exclusively the presence of IAPV. The extensive information obtain by a metagenomic approach is thus of great value when trying to understand multifactorial diseases, such as CCD. Considering the importance of honey bees in agriculture, it is also noteworthy that we could further establish the role of honeybees as vectors of pollen-borne viruses of plants by the detection and identification of the TuRSV. Although this initial study managed to identify both a novel subtype of bee-infecting ALPV and LSV not previously reported in Europe, more studies are called for in order to establish the normal genetic and microbiomic background in bees from different geographic regions. Such information would enable comparison between metagenomic profiles from apparent healthy bees and from diseased animals, in order to provide further novel insights into the complex diseases of the honeybee.
